# Artificial Intelligence Reshapes Creativity: A Multidimensional Evaluation

**DOI:** 10.1002/pchj.70042

**Published:** 2025-08-05

**Authors:** Chenchen Zhang, Yong Shao, Yuan Yuan, Wangbing Shen

**Affiliations:** ^1^ School of Philosophy Nanjing University Nanjing China; ^2^ School of Government Nanjing University Nanjing China; ^3^ School of Special Education Nanjing Normal University of Special Education Nanjing China; ^4^ School of Education Science Anhui Normal University Wuhu China; ^5^ School of Public Administration Hohai University Nanjing China

**Keywords:** artificial intelligence, creativity, evaluation, human–machine collaboration

## Abstract

Artificial intelligence (AI) is reshaping creativity by challenging its long‐held status as a uniquely human faculty. This study uses bibliometric analysis to reveal AI’s evolution from a passive instrument to an active co‐creator that amplifies human intuition and expands creative possibilities. We highlight how AI‐driven evaluative frameworks offer more objective, scalable, and inclusive assessments of creativity, disrupting bias‐prone traditional methods. Also, this transformation raises pressing ethical and legal concerns, particularly regarding authorship, intellectual property, and recognition of machine‐generated outputs. By mapping these tensions and opportunities, the study provides a critical foundation for rethinking creativity in the age of human–machine collaboration. Our findings point toward an urgent need for new conceptual models that align innovation with ethical and societal responsibility.

## 1 Introduction

The rapid advancement of artificial intelligence (AI) is reshaping creativity, often considered the highest form of human wisdom. Conceptually, AI encompasses many technologies, from deep learning to natural language processing and image recognition, with various definitions (Haenlein and Kaplan 2019; Truong and Papagiannidis 2022). The concept dates back to the 20th century, emerging in 1956 at a Dartmouth conference (Xu et al. 2021). Despite early setbacks, AI has grown exponentially, driven by systems intelligence (Russell 1997). This growth is anchored in six subfields: natural language processing, knowledge representation, automated reasoning, machine learning, computer vision, and robotics (Russell and Norvig 2016). In its broadest sense, AI refers to machines' capacity to emulate cognitive functions traditionally attributed to human intelligence (Tan 2019). Rai et al. (2019) described it as the ability of machines to engage in activities such as perception, reasoning, and problem‐solving. The New Oxford Dictionary (2019) defines AI as systems capable of performing tasks requiring human intelligence, including perception, speech recognition, decision‐making, and language translation. AI, powered by big data, digitalization, and advanced methods, is not only a technological breakthrough but also challenges the essence of human creativity.

Industries worldwide are rapidly recognizing the value of AI to spark a new wave of industrial transformation. From healthcare to automotive manufacturing, AI enables companies to reinvent their business models, automate complex processes, and drive efficiency at unprecedented scales (Iansiti and Lakhani 2020). The AI‐driven innovation ecosystem is expanding, with projections indicating that the global AI market will experience explosive growth over the next decade. We are witnessing more than a technological shift; AI is becoming a key driver of economic productivity and value creation. In this new landscape, creativity itself is being transformed, as AI is increasingly playing a role in the generation of ideas, solutions, and even artistic expression. A subset of AI, namely, machine learning, enables algorithms to learn autonomously from data and improve with experience. Together with digital sensors, interconnected networks, and software automation, AI is poised to drive a new era of industrialization—one in which the boundaries between human and machine creativity blur (Iansiti and Lakhani 2020). As Andrew Ng, a top AI expert who has led AI projects at Stanford, Google, and Baidu, noted, tasks that were once considered distinctly human—such as completing mental tasks within seconds—are now becoming ripe for automation through AI (Ng 2016).

AI has already demonstrated its capacity to influence a wide array of industries beyond simple task automation. It has been used by companies like Amazon to optimize supply chains and by Google's DeepMind to identify protein structures (Baek et al. 2021). AI has also generated solutions to complex problems and enabled groundbreaking innovations. This is particularly evident in industries where creative output is central to creativity, such as design, advertising, and entertainment (Huh et al. 2023; Wu and Jing Wen 2021). As AI continues to evolve, it will likely redefine how businesses, governments, and societies perceive and harness creative potential. This will lead to the need for new models of collaboration between humans and machines regarding creativity. Historically, creativity has been considered a uniquely human trait. However, AI is increasingly blurring the lines between human and machine creativity. As AI continues to evolve, its potential to augment human creativity represents one of the most exciting and challenging prospects of the 21st century. That is, creativity is no longer a solely human domain. The growing role of AI in creative work raises important questions about the future of creativity. As AI evolves, will creativity become shared or led by machines? Understanding the implications of AI for creativity is an urgent necessity for policymakers, educators, artists, and industry leaders.

The impact of AI on creativity is a critical area of contemporary research with profound implications for industries, society, and the concept of human ingenuity itself. AI is not only augmenting human capabilities, but also reshaping the very foundations of creativity. This is prompting us to fundamentally reevaluate what it means to be “creative.” In this study, we examine how AI is transforming the very essence of creativity. Our goal is to inspire new ways of thinking about the future of creative work, the role of technology in innovation, and the changing nature of human expression in an AI‐driven world. Taking a novel perspective on human–machine co‐creation, this study provides an in‐depth examination of the evolving relationship between AI and creativity. It offers a critical perspective on the role of AI in reshaping human‐ and machine‐driven innovation. Through this evaluation, which builds on bibliometric analyses, we hope to shed light on the emerging frontiers of creative work in an AI‐dominated future, and to stimulate further discussion about the implications of these changes for creative industries, economic growth, and wider society.

This article is structured as follows. First, we present a bibliometric analysis of literature on reshaping psychological creativity via AI. Second, we explore how AI is transforming the concept of creativity and challenging traditional views of human creativity. Third, we examine how AI is altering the creative process itself. Finally, we discuss the roles of AI in evaluating creativity and provide a concluding summary.

## 2 Bibliometric Analyses

### 2.1 Literature Search and Inclusion

To systematically assess the impact of AI on creativity, this study conducted a literature search within the SSCI+SCIE database of Web of Science (WOS) in June 2025. No specific time frame was restricted, only a deadline, in order to allow for an unimpeded, detailed depiction of the natural evolutionary trends in this field. The search used the topic or theme keywords “artificial intelligence” and “creativity,” with a focus on cognitive or psychological creativity research (WOS research areas), as creativity encompasses a wide range of domains. The search was limited to psychology articles in the WOS categories. A total of 129 entries were retrieved. After removing five editorials and one paper without an abstract, the researchers reviewed the abstracts of the remaining articles (*n* = 123) to determine the aspect of creativity influenced by AI. The data were analyzed via SPSS 25.0 software.

### 2.2 The Rational for Classifying Studies

Building on theoretical analysis and multiple rounds of group discussions, the following criteria were established for categorizing how AI reshapes creativity, spanning its conceptual, procedural, and evaluative dimensions:

1. The criteria for reshaping the essence of creativity: Studies that examine how AI challenges the uniqueness of human creativity, especially in fields like art, literature, and science, emphasize the conceptual shift in creativity. Other criteria include changes in creative behavior classification (e.g., blurring the lines between human‐generated and machine‐generated content) and ethical/legal issues related to AI in creative works.
2. The criteria for reshaping the process of creativity: Studies that highlight the roles of AI in the creative process, particularly in collaborative settings, are viewed as focusing on the transformation of creativity. Understanding AI as a guide, co‐creator, or contextual enabler is essential to grasping how the creative process is evolving.
3. The criteria for reshaping the assessment of creativity: the potential of AI to disrupt conventional evaluation models by offering global, real‐time, online, continuous, or non‐human‐centered assessments highlights its transformative impact on creativity evaluation.

### 2.3 Main Findings

**FIGURE 1:**
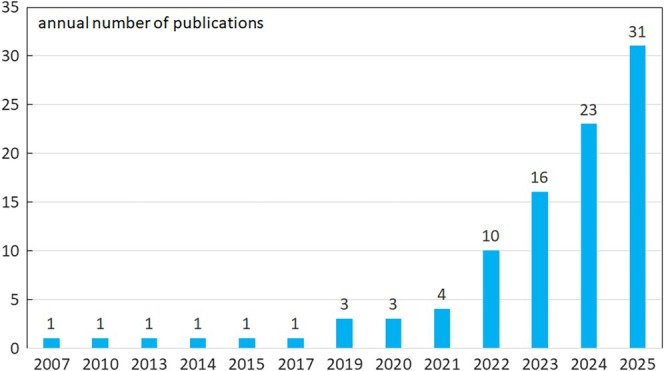
The (frequency) distribution of studies on the topic across different years.

Based on the criteria above, 27 studies were excluded from the original set of 123 articles because they did not fall into any of the three categories. The remaining 96 studies were further analyzed. Among them, 41 studies (42.70%) focused on the concept or essence of AI‐driven creativity; 19 studies (19.79%) addressed the reshaping of creativity evaluation; and 36 studies (37.50%) focused on the creative process. Of these 96 studies, 88 were classified as articles, while 8 were categorized as reviews. As shown in Figure 1, the earliest studies on AI reshaping creativity were published in 2007, and from 2019 onwards, at least three studies have been published annually. In 2025, 31 studies were identified, marking a significant increase, with the volume in 2024 being 23 studies, which is roughly 34% higher than the 2024 total. As illustrated in Table 1, the research on the role of AI in reshaping creativity spans a wide range of disciplines across WOS subject categories, with only eight studies from the SCI‐indexed database. One of the most prominent features of this field is its multidisciplinary nature. Also, contributions from educational psychology, experimental psychology, behavioral sciences, and computer science (with a specific focus on artificial intelligence) are especially prominent, highlighting the diverse scholarly perspectives engaged in this area of study.

**TABLE 1:** The distribution of target studies on creativity across the WOS categories.

Order	Times	WOS categories	Percentage
1	28	*Psychology, Multidisciplinary*	29.17
2	12	*Psychology, Educational*	12.50
3	9	*Humanities, Multidisciplinary; Psychology, Experimental*	9.38
4	9	*Psychology, Educational; Psychology, Multidisciplinary*	9.38
5	7	*Psychology, Experimental*	7.29
6	6	*Psychology, Multidisciplinary; Psychology, Experimental*	6.25
7	5	*Computer Science, Artificial Intelligence; Neurosciences; Psychology, Experimental*	5.21
8	4	*Psychology, Social*	4.17
9	3	*Behavioral Sciences; Psychology, Applied; Management; Psychology, Experimental*	3.13
10	3	*Business; Psychology, Applied*	3.13
11	1	*Engineering, Industrial; Ergonomics; Psychology, Applied; Psychology*	1.04
12	1	*Behavioral Sciences; Neurosciences; Psychology, Experimental*	1.04
13	1	*Humanities, Multidisciplinary; Psychology, Multidisciplinary*	1.04
14	1	*Linguistics; Psychology, Experimental*	1.04
15	1	*Music; Psychology, Experimental*	1.04
16	1	*Psychology, Applied*	1.04
17	1	*Psychology, Mathematical; Psychology, Experimental*	1.04

As shown in Table 2, 12 journals have contributed at least two studies to this field. Included in the list are four key journals in the field of creativity—*Journal of Creative Behavior*, *Journal of Intelligence*, *Creativity Research Journal*, and *Psychology of Aesthetics, Creativity and the Arts*—as well as *Computers in Human Behavior*, *Topics in Cognitive Science*, and *Cognitive Systems Research*, three influential journals on the application of computers in the behavioral or cognitive sciences. Furthermore, 24 other journals have each published a single study on the topic. They include *Acta Psychologia*, *American Psychologist*, *Annales Medico‐Psychologiques*, *Basic and Applied Social Psychology*, *Behavior Research Methods*, *Behavioral Sciences*, *Current Opinion in Behavioral Sciences*, *Empirical Studies of the Arts*, *Ergonomics*, *I‐Perception*, *Intelligence*, *Journal of Educational Psychology*, *Journal of Personality and Social Psychology*, *Mind & Language*, *Music Perception*, *Nature Human Behaviour*, *Personality and Social Psychology Bulletin*, *Psychology & Marketing, Perspectives on Psychological Science*, *Psychological Methods*, *Revista de Psicologia del Deporte*, *Social Cognitive and Affective Neuroscience*, *Social Behavior and Personality*, and *Cognitive Research‐Principles and Implications*.

**TABLE 2:** The list of major journals wherein the selected studies on creativity published (*n* ≥ 2).

Order	Source title	Times	Percentage
1	*Frontiers in Psychology*	14	14.58
2	*Journal of Creative Behavior*	11	11.46
3	*Creativity Research Journal*	9	9.38
4	*Psychology of Aesthetics, Creativity, and the Arts*	9	9.38
5	*Computers in Human Behavior*	6	6.25
6	*Journal of Intelligence*	6	6.25
7	*Cognitive Systems Research*	5	5.21
8	*Current Psychology*	3	3.13
9	*Journal of Applied Behavioral Science*	3	3.13
10	*Topics in Cognitive Science*	2	2.08
11	*Journal of Business and Psychology*	2	2.08
12	*Journal of Cognitive Psychology*	2	2.08

## 3 AI Reshapes the Essence of Creativity

AI is more than just a technological breakthrough; it is a transformative force redefining the very nature of human creativity. Recent applications of AI have prompted a reexamination of creativity in boundaries and essence. To explore any new concept scientifically, a clear definition is crucial (Shao et al. 2019; Zhao et al. 2022). However, defining creativity remains difficult due to its complexity and contested nature. Models like the 3P (Person, Process, and Product) and 4P (Person, Process, Product, and Press) frameworks emphasize the importance of “Person” and “Process” (Sasser and Koslow 2008; Gruszka and Tang 2017), reflecting common factors across various levels of creativity. While the “Person” dimension addresses traits such as agency, personality, motivation, and cognitive style, the “Process” dimension focuses more on the process of creative thinking (e.g., insight). These elements are central to human innovation and underscore the uniqueness of human creativity. Traditionally, creativity has been regarded as a distinctly human trait, a pinnacle of human intelligence. However, AI challenges this human‐centric view on creativity. This disruption manifests across four key dimensions: the unique status of human creativity, the transformation of creative collaboration, the evolving typology of creative acts, and the issue of copyright in creativity.

First, AI challenges the long‐standing belief that creativity is an exclusively human trait. Traditionally, creativity has been seen as inherently tied to human intuition, emotion, personal experience, and complex cognitive processes, reflecting the unique human condition. It is often defined as the ability to generate novel and meaningful ideas or works. However, the rise of generative AI technologies—such as GPT, DALL·E, DEEPSEEK, and DeepArt—has disrupted this view. These systems can now produce creative outputs, including short stories, music, mathematical solutions, and visual art, often indistinguishable from human masters (Dornis 2020; Köbis and Mossink 2021; Mazzone and Elgammal 2019). Our bibliometric analysis reveals that 42.7% of studies focus on the redefinition of creativity through the lens of artificial intelligence. This raises fundamental questions: Can machines be creative? Can AI generate creativity that is not merely derivative but revolutionary? Is creativity merely the result of human emotion and intentionality, or can it also emerge from vast datasets and algorithmic processes? AI is increasingly central to artistic domains, not just as a tool but as a cocreator in visual arts, music, and film. This prompts the critical question of whether *AI can possess its own form of creativity*—perhaps even one that complements or surpasses human creativity. Consequently, we must reconceptualize creativity as a dynamic, interspecies process, wherein humans and machines collaborate in cocreation.

Second, AI challenges traditional notions of human creativity by introducing a model of human–machine cocreation that expands and enhances creative agency (Dang and Liu 2025). Rather than replacing human creativity, AI acts as a collaborative partner, enabling humans to explore a broader range of possibilities, refine ideas, and develop them further. By generating multiple creative options rapidly, AI facilitates complex and diverse outcomes that go beyond linear thinking. For example, in music, AI can generate melodies or harmonies that serve as starting points for composers, opening new avenues for experimentation (Cole 2020). In design, AI tools like Autodesk are revolutionizing the process by producing prototypes aligned with predefined goals (Chandrasekera et al. 2025), such as brand identity or market trends. Additionally, by automating time‐intensive tasks such as programming or initial artwork (Gamez‐Djokic et al. 2025), the creative cycle is accelerated, enabling creators to concentrate on deeper, symbolic creations. The iterative feedback‐driven process fosters continuous experimentation, fueling innovation. In this way, AI becomes more than a tool; it transforms into an active engagement in the creative process, blurring the line between creators and instruments.

This evolution challenges traditional notions of creative agency, redefining the “creative actor.” Many creativity definitions (Boden 1998, 2004; Puryear and Lamb 2020) stress the generation of novel and valuable outputs, which AI fulfills. As AI produces results that meet established standards, it challenges notions of the “creative actor.” By simulating divergent thinking (Stevens Jr and Zabelina 2020) and problem‐solving (Colin et al. 2016), AI shifts from being a mere tool to a genuine collaborator, which redefines creativity as a collaborative process between humans and machines.

Third, the evolution of generative AI is rethinking creativity. Traditional models, such as the “4C” framework, position creativity from “mini‐C” (personal) to “big‐C” (exceptional). As AI progresses, it introduces new forms that blur these categories. Leverage AI to process vast datasets and rapidly generate creative solutions in fields such as drug discovery and materials science (e.g., Callaway 2022), accelerating innovation through the exploration of multiple scenarios. This can result in a “creative surge,” where AI‐driven insights reveal breakthroughs. While AI accelerates incremental advancements and supports expert‐level creativity, its capacity to catalyze radical societal change is unclear. This distinction highlights the contrast between incremental and transformative change. Ivcevic and Grandinetti (2024) explored how AI influences creativity across four levels—mini‐C, little‐C, Pro‐C, and Big‐C—and reported that AI tools vary in their ability to support specific types of creativity or span the entire spectrum. In parallel, as AI development continues, concepts like digital and computational creativity have emerged, broadening the scope of what creativity can encompass.

Finally, AI is challenging traditional notions of creativity, ownership, and attribution, raising the need to reconsider the ethical and legal implications of AI‐generated works. As AI becomes embedded in digital and networked platforms, questions arise about the ownership of its outputs. Should rights be granted to the developer, the user, or the AI itself? Current legal and cultural frameworks (Cohen 2017) for authorship may be inadequate to handle such cases, especially as AI‐generated content becomes more autonomous. These questions highlight the need for updated legal frameworks that address shifting boundaries of authorship. While AI can produce valuable outputs, they often lack the emotional depth, intent and social context inherent to human endeavors, raising debates over whether such works can be deemed “art” or “meaningful.” As AI plays a growing role in creative processes, these concerns take on greater significance, with far‐reaching implications for intellectual property law and the future of creative work. The potential of AI is tempered by ethical risks, especially the possibility of “unrestricted” development yielding unintended, potentially harmful outcomes (Shen et al. 2019). This is evident in the concept of malevolent creativity, which examines the capacity of AI to generate harmful content (Gao et al. 2022; Hunter et al. 2022). As AI systems gain autonomy, questions regarding the motivations behind their outputs and their evaluation become increasingly important. These issues demand a reassessment of creativity and the ethical and legal frameworks that govern it in an AI‐driven world.

To conclude, AI is revolutionizing the concept of creativity by challenging the belief that it is a unique human trait. Creativity is now being reconceptualized as a dynamic, collaborative process in which machines actively participate. AI can generate novel and valuable outputs in art, music, and scientific discovery, demonstrating its capacity to contribute creatively in ways once considered impossible. This change fosters a new model of collaboration, where humans and AI co‐create, expanding the range of creative possibilities. However, the involvement of AI also raises significant ethical and legal questions. As AI continues to evolve, creating independently, these challenges compel us to rethink the nature of creativity and its authorship. In this evolving landscape, AI is transforming creativity from a solitary human endeavor into an interdependent force, reshaping intellectual property and ethical standards.

## 4 AI Reshapes the Process of Creativity

The “Process” dimension of creativity emphasizes the procedural aspects, focusing on the steps and methods that underpin creative activities. Creativity usually unfolds through a series of structured and purposeful stages, from problem definition to idea generation, processing, and feedback. These stages are intricately linked, forming a dynamic cycle of cognitive operations based on existing representations, concepts, and symbols. Numerous theoretical models have sought to explain these processes, but none fully encapsulates the essence of creativity, leaving much of the process still enshrouded in the so‐called “black box” (Grilli and Pedota 2024). However, our bibliometric analysis shows that 33.3% of the literature explores how AI is reshaping the creative process, highlighting its transformative role in generating, refining, and evolving ideas. This shift is fundamentally changing creative practices across various fields, from the arts to science, expanding the boundaries of traditional creativity. Recent research identifies three key ways AI is driving this change: as a creative guide, a collaborative partner, and a force that redefines the environment in which creativity flourishes (e.g., Chandrasekera et al. 2025; Pearson 2023). In these roles, AI not only supports the generation of ideas but also reshapes the structures and conditions under which creativity emerges.

AI has become a transformative force in the creative process, acting as a powerful tutor that enhances and simplifies creative tasks. The generation‐evaluation model posits that creativity unfolds in two stages: idea generation and evaluation (Grilli and Pedota 2024). During the generation phase, AI produces preliminary concepts, which are refined through human collaboration. In the evaluation phase, AI predicts potential by comparing concepts against a database. This method mirrors human evaluation but offers a broader, data‐driven perspective. The Geneplore model highlights the value of AI in creativity by making diverse information accessible and allowing knowledge to be recombined (Chua et al. 2015; Shen et al. 2024; Plucker 2023). AI plays a crucial role in synthesizing and selecting information, guiding creators toward creative solutions (Bink and Marsh 2000).

This shift in the role of AI represents a fundamental change in creativity. AI recalibrates the creative process by addressing human biases, expanding perspectives, and challenging norms. Just as the internet and search engines have transformed the way we retrieve information, an impact known as the “Google effect” (Sparrow et al. 2011), the growing integration of AI into daily life is giving rise to a distinct “ChatGPT effect.” In this new era, AI emerges as a vital and creative partner in human endeavors (Essel et al. 2024). In this human–machine co‐creation model, human intuition and computational power converge, transforming creativity into a dual‐agent process. AI catalyzes innovation, refines ideation, and generates fresh insights, reshaping the boundaries of human creativity.

Second, AI has emerged as an interactive co‐creator, sharing creativity between humans and machines. Creativity is no longer exclusively human as AI can now generate original works with minimal human input. This shift marks a fundamental change in how creative processes unfold. AI can “reverse engineer” human creativity, simulating the iterative processes that characterize human innovation (Boden 2004; Mazzone and Elgammal 2019). AI‐generated art exemplifies this potential. For instance, the portrait “Edmond de Belamy” produced by a generative adversarial network (GAN) trained on 15,000 portraits, achieved a level of originality indistinguishable from human‐created art (Mazzone and Elgammal 2019). This raises critical questions about originality and challenges traditional boundaries in art. Within this framework, human intuition and philosophical insight converge with the computational power of AI, forming a dynamic partnership that broadens creative possibilities.

The emergence of the human–machine cocreation model fundamentally reshapes traditional creative processes, particularly in how humans interact with AI. As AI becomes more intelligent, creators must invest time and energy not only in guiding the AI but also in understanding its intentions and creative process, adding complexity to the collaboration. This effort is likely to increase as AI systems advance, unless AI eventually surpasses human intelligence, reducing the need for human input in certain creative domains. Furthermore, the advent of generative AI has shifted the focus from merely solving creative problems to posing creative questions. The act of framing new problems or viewing old problems from novel angles is now recognized as a critical aspect of creativity—an insight echoed by Albert Einstein's assertion that “the formulation of the problem is often more essential than its solution.” In this context, AI goes beyond problem‐solving to enable a question‐driven, exploratory approach to creativity. This not only broadens the scope of human imagination but also creates new pathways for innovation, marking a shift in the creative landscape (Grilli and Pedota 2024; Bouschery et al. 2023; Haefner et al. 2021).

Finally, AI is fundamentally reshaping the creative process by amplifying and accelerating human ingenuity. As AI becomes integral to creative workflows, frameworks such as Gruner and Csikszentmihalyi (2019) Creativity 4.0 model are gaining relevance, incorporating AI as a central player within the creative ecosystem. This reconceptualization emphasizes the role of AI in the interaction between the individual, domain, and AI‐enabled tools, shifting the internal dynamics that drive creativity. By augmenting human cognitive abilities with computational efficiency, AI fosters a more dynamic, rapid, and diverse approach to innovation, broadening the scope and depth of creative output. AI facilitates exploratory creativity, enabling creators to break free from conventional thinking and explore new possibilities. Through the rapid generation of numerous combinations within defined parameters, AI accelerates decision‐making and refines outcomes. For instance, AI can produce multiple design variations in a fraction of the time required by human creators, offering crucial insights that guide further exploration. Beyond its computational capabilities, AI personalizes creative content by analyzing vast datasets on user preferences, behaviors, and trends, enabling the production of highly tailored works that resonate with specific audiences—particularly evident in digital media, where AI designs personalized user experiences.

AI is reshaping creativity across various domains. It is a “creative enabler” transforming traditional creative workflows by altering the roles of key elements such as ideation, iteration, and feedback. This enhances the quality and speed of creative work (Grilli and Pedota 2024). AI provides creators with “heuristic maps” or “mental routes” and helps them build upon existing knowledge and navigate the creative journey more efficiently. Its ability to quickly generate and assess numerous possibilities allows creators to identify the most promising paths with greater precision. Its advanced data retrieval and analysis capabilities mitigate cognitive biases and narrow search patterns, facilitating a deeper exploration of novel challenges and fostering innovative solutions (Grilli and Pedota 2024). AI unlocks opportunities for groundbreaking, unconventional creative outputs, enabling creators to push the boundaries of their work (Bouschery et al. 2023; Haefner et al. 2021).

At the mechanistic level, the co‐creation process between humans and AI aligns with Dual‐Process Theory. Humans engage in heuristic processing (System 1), relying on intuition, emotion, and experience to direct and refine creativity. In contrast, AI operates through analytical processing (System 2), using data and algorithms to systematically generate and refine outputs. This dynamic interaction forms a feedback loop, where human input shapes AI's immediate responses and contributes to its future iterations, enhancing adaptability. However, we contend that AI's creativity remains programmatic, primarily limited to incremental and combinatorial creativity (Rafner et al. 2023). AI is not yet capable of achieving Pro‐c or big‐C creativity, which requires surprise, novelty, and non‐algorithmic, open‐ended tasks. Additionally, both humans and AI systems are prone to biases. AI's bias from training data can limit idea diversity (Doshi and Hauser 2024), affecting the inclusiveness of creative outputs. Nonetheless, the heterogeneous knowledge between humans and machines in co‐creation can significantly enhance the collaborative process. Insights from management psychology support this perspective. This collaboration often results in unexpected outcomes, where human intent and machine‐generated possibilities blend, producing novel creations that surpass both human expectations and AI's original algorithms. This synergy fosters innovation, leading to a creative process that is more expansive and impactful than either component could achieve independently (see Wu et al. 2021).

To conclude, AI is reshaping creativity. It is evolving from a mere tool to a collaborative partner. It facilitates, interacts, and influences. It automates repetitive tasks in combinatorial and exploratory creativity. This frees creators to focus on higher‐level innovation. It accelerates the creative process through diverse possibilities and heuristic insights. AI supports creativity (Wingström et al. 2024) and generates novel ideas. It drives breakthroughs and pushes aesthetic boundaries. This challenges traditional notions of creativity. It also enhances the connection between creators and audiences. However, as AI broadens creative possibilities, it also brings up ethical concerns. These include authorship disputes, data privacy, and the potential for misinformation. With AI assuming more creative roles, questions arise about the displacement of human labor, the value of human originality, and ownership of AI‐generated content. These concerns underscore the need for a more cautious and critical approach to understanding AI's impact on creativity. This is particularly regarding public trust, societal stability, and the responsible use of technology.

## 5 AI Reshapes the Assessment of Creativity

Assessing creativity remains a central yet challenging issue in creativity research, particularly due to concerns about subjectivity and the time‐consuming nature of traditional evaluation methods. As Plucker et al. (2019) noted, creativity can be assessed across multiple dimensions, referred to as the “Four Ps” by Rhodes (1961). Subjectivity arises from inconsistency among raters, while traditional evaluations require substantial resources (Beaty and Johnson 2021; Cropley and Marrone 2025). These challenges make creativity assessment complex and time‐consuming. However, advancements in AI, especially machine learning and natural language processing (Acar et al. 2025; Hussain et al. 2024; Agnoli and Mastria 2022), are facilitating more efficient and scalable approaches to evaluating creative output (Shen and Shao 2019; Patterson et al. 2024). These offer standardized, objective, intelligent, and scalable assessments for creativity (Shen and Shao 2019; Patterson et al. 2024).

AI can assess creativity more holistically by shifting from reductionist models to systems‐based approaches. Traditional assessments of creativity focus on traits like divergent thinking, relying on subjective human judgment and frameworks on creative outcomes, which often lack the ability to fully capture the dynamic nature of creative processes (Silvia et al. 2009; Long and Wang 2022). In contrast, AI‐driven systems assess creativity as a dynamic, interconnected process, reflecting the holistic and dynamic nature of creative thinking. Rooted in Gestalt psychology, this approach emphasizes the interconnectedness and flow of creative problem‐solving. AI models, such as those used in creative painting tasks by Patterson et al. (2024), evaluate both originality and compositional characteristics, offering a more integrated perspective on creativity. AI systems also provide real‐time feedback on the entire creative process, not just the final product (Silvia et al. 2009). This enables more nuanced and comprehensive evaluations, deepening our understanding of creativity by balancing novelty with familiarity (Wei et al. 2022).

Moreover, integrating evaluation into the creative process is a key advantage over AI, enabling real‐time, continuous assessment. Traditional assessments of creativity often involve a separation between “generation” and “evaluation” (Kleinmintz et al. 2019), which can create barriers to understanding the creative process. AI, however, allows for an interactive and integrated system where both assessment and creativity occur simultaneously (Wei et al. 2022). This integration of the “generation–evaluation cycle” helps bridge the gap between the generation and evaluation stages, fostering a more natural approach to creativity in real‐world settings. Such integration also enhances the ecological validity of creativity assessments, particularly in educational environments (Agnoli and Mastria 2022), where creativity is nurtured through ongoing feedback and development.

Furthermore, AI is gradually influencing creativity assessment and driving significant transformation, reshaping traditional perspectives and methods. As noted by Cropley and Marrone (2025), the use of AI in creativity research has unlocked new possibilities in academic studies and practical fields like education. For example, an AI‐driven creativity assessment system based on computer vision was developed by Patterson et al. (2024) to evaluate creative drawings made by participants. The system, trained using a modified ResNet architecture, was shown to be capable of evaluating abstract and realistic sketches in real time, predicting their creativity levels with remarkable accuracy. Additionally, AI offers new evidence supporting a more universal view of creativity (Agnoli and Mastria 2022). This reframing allows for a more inclusive understanding of creativity.

Another profound effect of AI on creativity assessment is its potential to decenter human‐centric views of creativity, challenging the traditional belief that creativity is a uniquely human trait. As mentioned above, AI challenges this anthropocentric framework, indicating that creativity is not exclusive to humans but can also be manifested in machines. As Moruzzi (2021) noted, broadening the definition of creativity beyond human‐centered views is essential for a more inclusive and unbiased understanding of the concept. Evaluating creativity from a nonanthropocentric perspective enables new insights into controversial creative cases and helps dismantle entrenched biases, such as concerns over AI replacing human creativity (Chymis 2020; Caporusso 2023) and efforts to reinforce human‐centric notions (Köbis and Mossink 2021; Jia et al. 2024; Tigre Moura et al. 2023).

Taken together, AI is transforming creativity assessment through a more integrated, system‐based approach, enabling objective, inclusive, and real‐time evaluations. By merging generation with evaluation in a dynamic feedback loop, AI mirrors the iterative nature of creativity, offering continuous insights, especially in educational settings. This challenges anthropocentric views, broadening creativity to include both human and machine contributions. To enhance inclusivity and global relevance, AI must incorporate diverse cultural contexts, addressing traditional biases. However, challenges persist. Cross‐cultural studies show that creativity is shaped by cultural context, and AI models may overlook these subtleties (Henriksen et al. 2025). For instance, collectivist cultures often value works emphasizing community and social harmony, which AI models trained on individualistic outputs may miss. Embedding cultural sensitivity and contextual awareness into evaluation models is crucial to ensure AI reflects the values and norms of diverse societies (Jordanous and Keller 2016).

## 6 Concluding Remarks

AI is changing how we understand creativity. It is not just about what we can do, but how we work together to create. This is a big change that is happening in three ways: how we think about creativity, how we do creative things, and how we judge creativity. This study looks at all of these things and how they are changing because of AI.

AI amplifies human capabilities, introducing new dynamics to the creative process. AI excels at processing large datasets, generating diverse creative options, and reducing bias. However, it lacks the ability for intuitive, emotional, or radical creativity. While AI can offer a wide range of ideas and solutions, it cannot provide the deep, context‐driven insights or emotional expression needed for truly groundbreaking or emotionally impactful work (Zhang et al. 2025). Future research should explore optimal frameworks for human–AI collaboration.

No study is without limitations, and ours is no exception. While our research focuses on the WOS database, which includes leading international psychology journals, we recognize the exclusion of other valuable sources such as Scopus and PubMed. This deliberate focus on mainstream psychological perspectives could be expanded in future work to incorporate underrepresented interdisciplinary and empirical studies. Additionally, our study takes a mid‐level perspective, focusing on how AI influences creativity rather than delving into the complex cognitive mechanisms behind this influence. While we recognize the importance of exploring these mechanisms in more detail, our main goal is to offer a broad conceptual framework that connects AI and creativity, with further research needed to explore the specific cognitive dynamics. Furthermore, our study does not fully address the ethical and societal issues surrounding AI, such as authorship disputes and data privacy, which merit deeper exploration. For instance, generative models enhance individual creativity but may risk diminishing collective novelty, a dilemma that underscores the need for careful ethical consideration.

Despite the potential to augment creativity, there remain significant limitations compared to human creativity. AI's reliance on recombining existing data rather than generating truly novel insights means it often produces what Marcus and Davis (2019) describe as “plausible pastiche”—coherent but derivative outputs. A lack of subjective experience and emotional depth prevents the replication of the meaning‐making intrinsic to human creativity (Boden 2004), which drives cultural innovation. Moreover, overreliance on AI tools risks eroding human creative faculties by replacing essential learning processes with instant outputs, a phenomenon Shneiderman (2022) terms “creativity atrophy.” This is particularly concerning in educational contexts, where the use of AI may inhibit the development of critical thinking and imaginative skills. Additionally, legal uncertainties surrounding AI‐generated content—such as copyright and data provenance issues—create new constraints for human creators, potentially stifling experimentation. While AI undoubtedly plays a role in enhancing creative processes, its growing influence raises structural challenges to the authentic evolution of human creativity, warranting deeper investigation into its ethical implications and ontological potential.

## Conflicts of Interest

The authors declare no conflicts of interest.

## Data Availability

Data sharing not applicable to this article as no datasets were generated or analysed during the current study.
